# 1-{(*E*)-[4-Bromo-2-(trifluoro­meth­oxy)phen­yl]imino­meth­yl}naphthalen-2-ol

**DOI:** 10.1107/S160053681300679X

**Published:** 2013-03-16

**Authors:** Hakan Kargılı, Mustafa Macit, Gökhan Alpaslan, Orhan Büyükgüngör, Ahmet Erdönmez

**Affiliations:** a Department of Physics, Faculty of Arts & Science, Ondokuz Mayıs University, TR-55139 Kurupelit-Samsun, Turkey; b Department of Chemistry, Faculty of Arts & Science, Ondokuz Mayıs University, TR-55139 Kurupelit-Samsun, Turkey; c Department of Medical Services and Techniques, Vocational School of Health Services, Giresun University, TR-28200 Giresun, Turkey

## Abstract

The title compound, C_18_H_11_BrF_3_NO_2_, crystallizes in the phenol–imine tautomeric form, with a strong intra­molecular O—H⋯N hydrogen bond. The dihedral angle between the naphthalene ring system and the benzene ring is 28.54 (10)°.

## Related literature

For biological properties of Schiff bases, see: Layer (1963 ▶); Ingold (1969 ▶); Barton & Ollis (1979 ▶). For Schiff base tautom­erism, see: Hökelek *et al.* (2000 ▶); Tüfekçi *et al.* (2009 ▶). For related strucures, see: Bingöl Alpaslan *et al.* (2010 ▶); Soydemir *et al.* (2011 ▶).
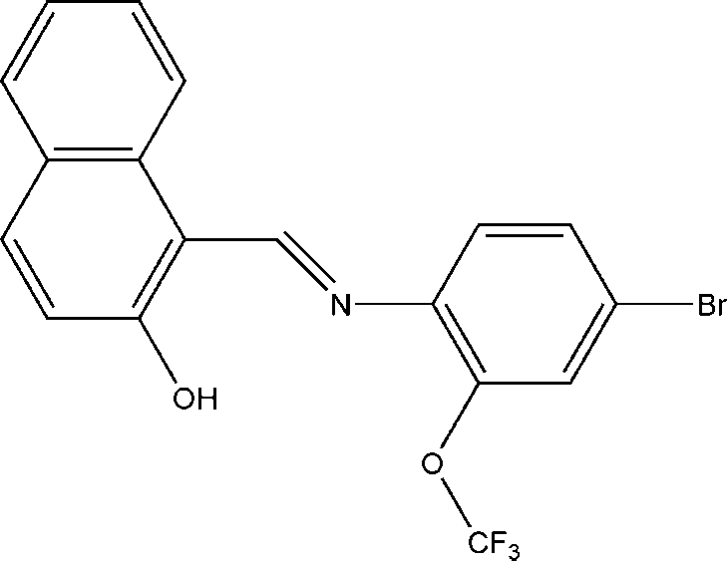


## Experimental

### 

#### Crystal data

C_18_H_11_BrF_3_NO_2_
*M* *_r_* = 410.19
Monoclinic, 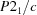

*a* = 4.5315 (3) Å
*b* = 16.3228 (7) Å
*c* = 21.7622 (12) Å
β = 93.025 (4)°
*V* = 1607.44 (15) Å^3^
*Z* = 4
Mo *K*α radiation
μ = 2.60 mm^−1^
*T* = 296 K
0.73 × 0.32 × 0.08 mm

#### Data collection

Stoe IPDS-II diffractometer
Absorption correction: integration (*X-RED32*; Stoe & Cie, 2002 ▶) *T*_min_ = 0.176, *T*_max_ = 0.772
17993 measured reflections
3160 independent reflections
1932 reflections with *I* > 2σ(*I*)
*R*_int_ = 0.080

#### Refinement

*R*[*F* ^2^ > 2σ(*F* ^2^)] = 0.050
*wR*(*F* ^2^) = 0.092
*S* = 1.02
3160 reflections
230 parameters
H atoms treated by a mixture of independent and constrained refinement
Δρ_max_ = 0.35 e Å^−3^
Δρ_min_ = −0.20 e Å^−3^

### 

Data collection: *X-AREA* (Stoe & Cie, 2002 ▶); cell refinement: *X-AREA*; data reduction: *X-RED32* (Stoe & Cie, 2002 ▶); program(s) used to solve structure: *SHELXS97* (Sheldrick, 2008 ▶); program(s) used to refine structure: *SHELXL97* (Sheldrick, 2008 ▶); molecular graphics: *ORTEP-3 for Windows* (Farrugia, 2012 ▶); software used to prepare material for publication: *WinGX* (Farrugia, 2012 ▶).

## Supplementary Material

Crystal structure: contains datablock(s) I, global. DOI: 10.1107/S160053681300679X/bg2500sup1.cif

Structure factors: contains datablock(s) I. DOI: 10.1107/S160053681300679X/bg2500Isup2.hkl

Supplementary material file. DOI: 10.1107/S160053681300679X/bg2500Isup3.cml

Additional supplementary materials:  crystallographic information; 3D view; checkCIF report

## Figures and Tables

**Table 1:** Hydrogen-bond geometry (Å, °)

*D*—H⋯*A*	*D*—H	H⋯*A*	*D*⋯*A*	*D*—H⋯*A*
O1—H1⋯N1	0.85 (5)	1.77 (6)	2.551 (5)	151 (5)

**Fig. 1.**
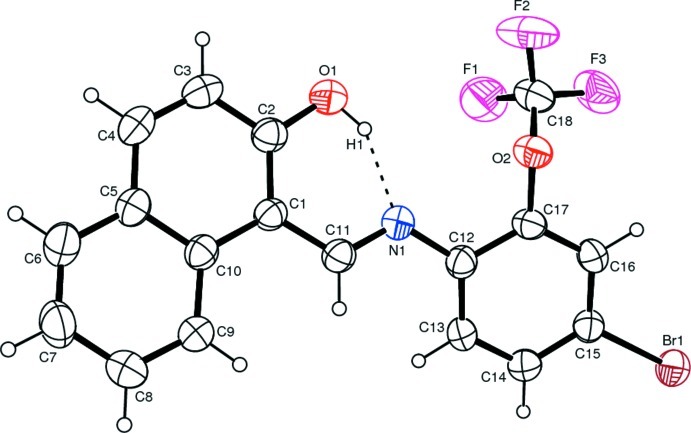
The molecular structure of (I), showing the atom-numbering scheme. Displacement ellipsoids are drawn at the 30% probability.

## supplementary crystallographic information

### Comment

Schiff bases are used as starting materials in the synthesis of important drugs, such as antibiotics and antiallergic, antiphlogistic, and antitumor substance (Layer 1963; Ingold 1969; Barton & Ollis, 1979). There are two types of intramolecular hydrogen bonds in Schiff bases, namely N—H···O in keto (NH) (Hökelek *et al.*, 2000) and N···H—O in enol (OH) (Tüfekçi *et al.*, 2009) tautomeric forms. The present X-ray investigation shows that the title compound is a Schiff base and exists in the phenol-imine form in the solid-state. An ORTEP-3 (Farrugia, 2012) plot of the molecule of (I) is shown in Fig.1. The C2-O1 bond length of 1.335 (5) Å indicates single-bond character while the N1-C11 bond length of 1.284 (4) Å indicates double-bond character.These bond distances are comparable with those of compounds previously reported as phenol-imine (Bingöl Alpaslan *et al.*, 2010; Soydemir *et al.*, 2011). The dihedral angle between the naphthalene ring system and the benzene ring is 28.54 (10)°.

### Experimental

(E)-1-[(4-bromo-2-(trifluoromethoxy)phenylimino)methyl]naphthalen-2-ol was prepared by refluxing a mixture of a solution containing 2-hydroxy-1-naphthaldehyde (17,22 mg, 0,1 mmol) in ethanol (20 ml) and a solution containing 4-bromo-2-(trifluoromethoxy)aniline (25,60 mg, 0,1 mmol) in ethanol (20 ml). The reaction mixture was stirred for 5 hour under reflux. Single crystals of the title compound for x-ray analysis were obtained by slow evaporation of an ethanol solution (Yield 68%; m.p. 376 - 378 K).

### Refinement

The H atom bonded to O1 was refined freely. All other H atoms were placed in calculated positions and constrained to ride on their parent atoms, with C–H = 0.93Å and *U*_iso_(H) = 1.2 *U*_eq_(C).

### Crystal data

**Table:**

C_18_H_11_BrF_3_NO_2_	*F*(000) = 816
*M**_r_* = 410.19	*D*_x_ = 1.695 Mg m^−^^3^
Monoclinic, *P*2_1_/*c*	Mo *K*α radiation, λ = 0.71073 Å
Hall symbol: -P 2ybc	Cell parameters from 16693 reflections
*a* = 4.5315 (3) Å	θ = 1.6–27.3°
*b* = 16.3228 (7) Å	µ = 2.60 mm^−^^1^
*c* = 21.7622 (12) Å	*T* = 296 K
β = 93.025 (4)°	Needle, yellow
*V* = 1607.44 (15) Å^3^	0.73 × 0.32 × 0.08 mm
*Z* = 4	

### Data collection

**Table:**

Stoe IPDS-II diffractometer	3160 independent reflections
Radiation source: fine-focus sealed tube	1932 reflections with *I* > 2σ(*I*)
Graphite monochromator	*R*_int_ = 0.080
Detector resolution: 6.67 pixels mm^-1^	θ_max_ = 26.0°, θ_min_ = 1.6°
ω scans	*h* = −5→5
Absorption correction: integration (*X-RED32*; Stoe & Cie, 2002)	*k* = −20→20
*T*_min_ = 0.176, *T*_max_ = 0.772	*l* = −26→26
17993 measured reflections	

### Refinement

**Table:**

Refinement on *F*^2^	Primary atom site location: structure-invariant direct methods
Least-squares matrix: full	Secondary atom site location: difference Fourier map
*R*[*F*^2^ > 2σ(*F*^2^)] = 0.050	Hydrogen site location: inferred from neighbouring sites
*wR*(*F*^2^) = 0.092	H atoms treated by a mixture of independent and constrained refinement
*S* = 1.02	*w* = 1/[σ^2^(*F*_o_^2^) + (0.0355*P*)^2^] where *P* = (*F*_o_^2^ + 2*F*_c_^2^)/3
3160 reflections	(Δ/σ)_max_ < 0.001
230 parameters	Δρ_max_ = 0.35 e Å^−^^3^
0 restraints	Δρ_min_ = −0.20 e Å^−^^3^

### Special details

**Table:**

Geometry. All esds (except the esd in the dihedral angle between two l.s. planes) are estimated using the full covariance matrix. The cell esds are taken into account individually in the estimation of esds in distances, angles and torsion angles; correlations between esds in cell parameters are only used when they are defined by crystal symmetry. An approximate (isotropic) treatment of cell esds is used for estimating esds involving l.s. planes.
Refinement. Refinement of F^2^ against ALL reflections. The weighted R-factor wR and goodness of fit S are based on F^2^, conventional R-factors R are based on F, with F set to zero for negative F^2^. The threshold expression of F^2^ > 2sigma(F^2^) is used only for calculating R-factors(gt) etc. and is not relevant to the choice of reflections for refinement. R-factors based on F^2^ are statistically about twice as large as those based on F, and R- factors based on ALL data will be even larger.

### Fractional atomic coordinates and isotropic or equivalent isotropic displacement parameters (Å^2^)

**Table:**

	*x*	*y*	*z*	*U*_iso_*/*U*_eq_	
C1	1.2279 (8)	0.2342 (2)	0.35440 (16)	0.0573 (9)	
C2	1.2003 (8)	0.3194 (3)	0.35626 (18)	0.0655 (10)	
C3	1.3639 (10)	0.3657 (3)	0.4008 (2)	0.0776 (11)	
H3	1.3428	0.4223	0.4018	0.093*	
C4	1.5515 (9)	0.3281 (3)	0.44213 (19)	0.0765 (11)	
H4	1.6587	0.3596	0.4710	0.092*	
C5	1.5892 (8)	0.2423 (3)	0.44265 (17)	0.0657 (10)	
C6	1.7837 (9)	0.2042 (3)	0.48585 (19)	0.0812 (12)	
H6	1.8893	0.2362	0.5147	0.097*	
C7	1.8217 (10)	0.1228 (4)	0.4867 (2)	0.0936 (14)	
H7	1.9523	0.0986	0.5157	0.112*	
C8	1.6626 (11)	0.0748 (3)	0.4434 (2)	0.0923 (14)	
H8	1.6867	0.0182	0.4441	0.111*	
C9	1.4735 (9)	0.1092 (3)	0.40055 (19)	0.0777 (11)	
H9	1.3718	0.0759	0.3720	0.093*	
C10	1.4281 (8)	0.1944 (2)	0.39847 (17)	0.0602 (9)	
C11	1.0578 (8)	0.1889 (2)	0.30832 (17)	0.0622 (9)	
H11	1.0735	0.1321	0.3078	0.075*	
C12	0.7139 (8)	0.1804 (2)	0.22366 (16)	0.0597 (9)	
C13	0.6122 (9)	0.1007 (3)	0.23012 (17)	0.0684 (10)	
H13	0.6661	0.0716	0.2657	0.082*	
C14	0.4340 (8)	0.0637 (3)	0.18521 (17)	0.0693 (10)	
H14	0.3668	0.0104	0.1906	0.083*	
C15	0.3558 (8)	0.1062 (2)	0.13217 (17)	0.0632 (10)	
C16	0.4535 (8)	0.1847 (2)	0.12336 (17)	0.0648 (10)	
H16	0.4010	0.2129	0.0873	0.078*	
C17	0.6300 (8)	0.2206 (2)	0.16873 (18)	0.0609 (9)	
C18	0.5876 (11)	0.3632 (3)	0.1640 (2)	0.0827 (12)	
Br1	0.11465 (10)	0.05593 (3)	0.06926 (2)	0.08462 (18)	
F1	0.4514 (8)	0.36567 (18)	0.21522 (15)	0.1282 (11)	
F2	0.7514 (7)	0.42801 (17)	0.16068 (19)	0.1371 (12)	
F3	0.3769 (6)	0.36943 (18)	0.11946 (14)	0.1162 (9)	
N1	0.8849 (7)	0.22428 (19)	0.26788 (14)	0.0653 (8)	
O1	1.0213 (7)	0.36180 (19)	0.31728 (16)	0.0838 (9)	
O2	0.7558 (6)	0.29790 (17)	0.15836 (12)	0.0725 (7)	
H1	0.963 (11)	0.327 (3)	0.290 (2)	0.12 (2)*	

### Atomic displacement parameters (Å^2^)

**Table:**

	*U*^11^	*U*^22^	*U*^33^	*U*^12^	*U*^13^	*U*^23^
C1	0.062 (2)	0.055 (2)	0.055 (2)	−0.0034 (18)	0.0068 (18)	−0.0039 (17)
C2	0.069 (2)	0.063 (3)	0.065 (3)	0.002 (2)	0.009 (2)	−0.004 (2)
C3	0.089 (3)	0.058 (3)	0.086 (3)	−0.003 (2)	0.007 (2)	−0.015 (2)
C4	0.080 (3)	0.077 (3)	0.072 (3)	−0.012 (2)	−0.002 (2)	−0.018 (2)
C5	0.064 (2)	0.073 (3)	0.060 (2)	−0.003 (2)	0.0049 (19)	−0.008 (2)
C6	0.076 (3)	0.095 (4)	0.071 (3)	0.004 (3)	−0.007 (2)	−0.011 (2)
C7	0.093 (3)	0.109 (4)	0.077 (3)	0.021 (3)	−0.020 (2)	0.000 (3)
C8	0.117 (4)	0.070 (3)	0.088 (3)	0.015 (3)	−0.008 (3)	0.007 (3)
C9	0.099 (3)	0.063 (3)	0.069 (3)	0.006 (2)	−0.010 (2)	−0.006 (2)
C10	0.063 (2)	0.064 (3)	0.054 (2)	−0.0020 (19)	0.0071 (17)	−0.0050 (18)
C11	0.070 (2)	0.059 (3)	0.058 (2)	0.0022 (19)	0.0080 (19)	−0.0040 (19)
C12	0.063 (2)	0.062 (3)	0.054 (2)	0.0051 (19)	0.0022 (18)	−0.0035 (19)
C13	0.090 (3)	0.064 (3)	0.051 (2)	−0.005 (2)	−0.004 (2)	0.0055 (19)
C14	0.082 (2)	0.059 (2)	0.066 (2)	−0.008 (2)	0.0015 (19)	0.000 (2)
C15	0.069 (2)	0.064 (3)	0.056 (2)	0.0049 (19)	−0.0028 (18)	−0.0029 (19)
C16	0.069 (2)	0.065 (3)	0.059 (2)	0.004 (2)	−0.0098 (18)	0.007 (2)
C17	0.062 (2)	0.052 (2)	0.069 (2)	0.0020 (18)	0.0004 (19)	0.0051 (19)
C18	0.086 (3)	0.064 (3)	0.097 (4)	0.004 (3)	0.001 (3)	0.017 (3)
Br1	0.0931 (3)	0.0795 (3)	0.0783 (3)	−0.0037 (3)	−0.02406 (19)	−0.0046 (3)
F1	0.157 (3)	0.111 (2)	0.120 (2)	0.0348 (19)	0.034 (2)	−0.0057 (19)
F2	0.119 (2)	0.0595 (18)	0.230 (4)	−0.0058 (17)	−0.011 (2)	0.026 (2)
F3	0.1036 (19)	0.107 (2)	0.134 (2)	0.0219 (16)	−0.0291 (17)	0.0330 (18)
N1	0.0728 (19)	0.063 (2)	0.0594 (19)	0.0005 (16)	−0.0006 (16)	−0.0015 (16)
O1	0.103 (2)	0.063 (2)	0.084 (2)	0.0087 (17)	−0.0090 (18)	−0.0022 (17)
O2	0.0727 (16)	0.0599 (18)	0.0845 (19)	0.0017 (15)	0.0006 (14)	0.0078 (14)

### Geometric parameters (Å, º)

**Table:**

C1—C2	1.397 (5)	C11—N1	1.284 (4)
C1—C11	1.437 (5)	C11—H11	0.9300
C1—C10	1.440 (5)	C12—C13	1.390 (5)
C2—O1	1.335 (5)	C12—C17	1.399 (5)
C2—C3	1.409 (6)	C12—N1	1.401 (5)
C3—C4	1.352 (6)	C13—C14	1.375 (5)
C3—H3	0.9300	C13—H13	0.9300
C4—C5	1.411 (6)	C14—C15	1.377 (5)
C4—H4	0.9300	C14—H14	0.9300
C5—C6	1.400 (6)	C15—C16	1.372 (5)
C5—C10	1.412 (5)	C15—Br1	1.893 (4)
C6—C7	1.339 (6)	C16—C17	1.369 (5)
C6—H6	0.9300	C16—H16	0.9300
C7—C8	1.396 (6)	C17—O2	1.408 (4)
C7—H7	0.9300	C18—F2	1.297 (5)
C8—C9	1.356 (6)	C18—F1	1.302 (5)
C8—H8	0.9300	C18—O2	1.320 (5)
C9—C10	1.406 (5)	C18—F3	1.329 (5)
C9—H9	0.9300	O1—H1	0.85 (5)
			
C2—C1—C11	119.1 (3)	N1—C11—C1	122.2 (4)
C2—C1—C10	118.9 (3)	N1—C11—H11	118.9
C11—C1—C10	121.9 (3)	C1—C11—H11	118.9
O1—C2—C1	123.3 (4)	C13—C12—C17	116.7 (3)
O1—C2—C3	116.1 (4)	C13—C12—N1	125.7 (3)
C1—C2—C3	120.6 (4)	C17—C12—N1	117.5 (4)
C4—C3—C2	120.3 (4)	C14—C13—C12	121.7 (4)
C4—C3—H3	119.9	C14—C13—H13	119.2
C2—C3—H3	119.9	C12—C13—H13	119.2
C3—C4—C5	121.9 (4)	C13—C14—C15	119.3 (4)
C3—C4—H4	119.1	C13—C14—H14	120.3
C5—C4—H4	119.1	C15—C14—H14	120.3
C6—C5—C4	121.2 (4)	C16—C15—C14	121.2 (4)
C6—C5—C10	119.7 (4)	C16—C15—Br1	118.8 (3)
C4—C5—C10	119.0 (4)	C14—C15—Br1	120.0 (3)
C7—C6—C5	121.7 (4)	C17—C16—C15	118.7 (4)
C7—C6—H6	119.1	C17—C16—H16	120.7
C5—C6—H6	119.1	C15—C16—H16	120.7
C6—C7—C8	119.1 (4)	C16—C17—C12	122.5 (4)
C6—C7—H7	120.5	C16—C17—O2	119.6 (3)
C8—C7—H7	120.5	C12—C17—O2	117.6 (3)
C9—C8—C7	121.1 (5)	F2—C18—F1	108.7 (5)
C9—C8—H8	119.5	F2—C18—O2	108.6 (4)
C7—C8—H8	119.5	F1—C18—O2	114.1 (4)
C8—C9—C10	121.3 (4)	F2—C18—F3	106.7 (4)
C8—C9—H9	119.4	F1—C18—F3	105.5 (4)
C10—C9—H9	119.4	O2—C18—F3	113.0 (4)
C9—C10—C5	117.1 (4)	C11—N1—C12	122.4 (3)
C9—C10—C1	123.6 (4)	C2—O1—H1	104 (4)
C5—C10—C1	119.3 (4)	C18—O2—C17	117.9 (3)
			
C11—C1—C2—O1	0.3 (5)	C2—C1—C11—N1	1.4 (5)
C10—C1—C2—O1	179.8 (3)	C10—C1—C11—N1	−178.1 (3)
C11—C1—C2—C3	−180.0 (3)	C17—C12—C13—C14	−1.0 (5)
C10—C1—C2—C3	−0.5 (5)	N1—C12—C13—C14	177.0 (3)
O1—C2—C3—C4	−179.8 (4)	C12—C13—C14—C15	0.6 (6)
C1—C2—C3—C4	0.5 (6)	C13—C14—C15—C16	0.2 (5)
C2—C3—C4—C5	−0.5 (6)	C13—C14—C15—Br1	179.2 (3)
C3—C4—C5—C6	−179.8 (4)	C14—C15—C16—C17	−0.5 (5)
C3—C4—C5—C10	0.5 (6)	Br1—C15—C16—C17	−179.5 (3)
C4—C5—C6—C7	−179.9 (4)	C15—C16—C17—C12	−0.1 (5)
C10—C5—C6—C7	−0.1 (6)	C15—C16—C17—O2	174.0 (3)
C5—C6—C7—C8	−0.2 (7)	C13—C12—C17—C16	0.8 (5)
C6—C7—C8—C9	0.6 (7)	N1—C12—C17—C16	−177.4 (3)
C7—C8—C9—C10	−0.7 (7)	C13—C12—C17—O2	−173.3 (3)
C8—C9—C10—C5	0.4 (6)	N1—C12—C17—O2	8.4 (5)
C8—C9—C10—C1	−179.3 (4)	C1—C11—N1—C12	−178.6 (3)
C6—C5—C10—C9	0.0 (5)	C13—C12—N1—C11	27.1 (5)
C4—C5—C10—C9	179.8 (4)	C17—C12—N1—C11	−154.8 (3)
C6—C5—C10—C1	179.8 (3)	F2—C18—O2—C17	171.8 (4)
C4—C5—C10—C1	−0.5 (5)	F1—C18—O2—C17	50.4 (6)
C2—C1—C10—C9	−179.8 (3)	F3—C18—O2—C17	−70.0 (5)
C11—C1—C10—C9	−0.3 (5)	C16—C17—O2—C18	79.8 (5)
C2—C1—C10—C5	0.5 (5)	C12—C17—O2—C18	−105.9 (4)
C11—C1—C10—C5	180.0 (3)		

### Hydrogen-bond geometry (Å, º)

**Table:**

*D*—H···*A*	*D*—H	H···*A*	*D*···*A*	*D*—H···*A*
O1—H1···N1	0.85 (5)	1.77 (6)	2.551 (5)	151 (5)
